# Clinical efficacy of acupuncture for pain relief from renal colic: A meta-analysis and trial sequence analysis

**DOI:** 10.3389/fmed.2022.1100014

**Published:** 2023-01-09

**Authors:** Hsiao-Tien Chen, Cheng-Feng Kuo, Chin-Chia Hsu, Li-Chun Lai, Ai-Chin Cheng, Cheuk-Kwan Sun, Kuo-Chuan Hung

**Affiliations:** ^1^Department of Chinese Medicine, Chi Mei Medical Center, Tainan City, Taiwan; ^2^Department of Pharmacy, Chi Mei Medical Center, Tainan City, Taiwan; ^3^Department of Nursing, Chi Mei Medical Center, Tainan City, Taiwan; ^4^Division of Respiratory Therapy, Department of Internal Medicine, Chi Mei Medical Center, Tainan City, Taiwan; ^5^Department of Medical Sociology and Health Care Bachelor’s Degree Program, College of Health Sciences, Chang Jung Christian University, Tainan City, Taiwan; ^6^Department of Emergency Medicine, E-Da Hospital, I-Shou University, Kaohsiung City, Taiwan; ^7^School of Medicine for International Students, College of Medicine, I-Shou University, Kaohsiung City, Taiwan; ^8^Department of Anesthesiology, Chi Mei Medical Center, Tainan City, Taiwan

**Keywords:** acupuncture, renal colic, meta-analysis, pain, trial sequence analysis

## Abstract

**Background:**

This meta-analysis aimed at investigating the efficacy of acupuncture for relieving renal colic and reducing the risk of analgesic-related complications.

**Methods:**

Randomized controlled trials (RCTs) comparing the efficacy of acupuncture (acupuncture group) with conventional interventions (control group) were screened from MEDLINE, EMBASE, Cochrane library databases, China Knowledge Network (CNKI), and Airiti Library till July 15, 2022. The primary outcome was the rate of effective pain relief (response rate), while secondary outcomes included the time of onset of pain relief, visual analog scale (VAS) at 30–60 min and risk of side effects.

**Results:**

Thirteen eligible studies involving 1,212 participants published between 1992 and 2021 were analyzed. Compared with the control group, patients receiving acupuncture had a higher overall response rate [risk ratio (RR) = 1.12, 95% CI: 1.05–1.19, *p* = 0.0002, *I*^2^ = 41%, 1,136 patients] (primary outcome) and a faster pain relief [MD = −10.74 min, 95% CI: −12.65 to −8.82, *p* < 0.00001, *I*^2^ = 87%, 839 patients]. Patients receiving acupuncture had a lower pain score [MD = −0.65, 95% CI: −1.09 to −0.21, *p* = 0.21, *I*^2^ = 55%, 327 patients] and risk of side effects (RR = 0.11, 95% CI: 0.04–0.26, *p* < 0.00001, *I*^2^ = 0, 314 patients) compared to those receiving conventional interventions. Results from trial sequence analysis revealed sufficient evidence supporting the beneficial effects of acupuncture on response rate, time to pain relief, and pain score at 30–60 min.

**Conclusion:**

Compared with conventional analgesic-based interventions, acupuncture can more efficiently relieve renal colic with fewer adverse effects. The limited number and quality of included studies warrant more clinical RCTs to support our findings.

**Systematic review registration:**

https://www.crd.york.ac.uk/prospero/, identifier CRD42022346714.

## 1. Introduction

Renal colic, which usually presents as a sudden-onset, sharp, paroxysmal abdominal pain in the lower back or upper abdomen that may extend from the ureter to the groin, inner thighs, and perineum (1), accounts for 12% of total hospitalization in the United States (2). The lifetime risk of renal colic is as high as 10–15% (3, 4). Severe pain from renal colic, which is the main reason for emergency department visits for those with the condition (5), origins from ureter obstruction and distension that cause reflexive peristaltic smooth muscle spasm, which leads to intensive visceral pain transmitted through the ureteric plexus (6). The recommended first-line treatment is non-steroidal anti-inflammatory drugs (NSAIDs) (7), opioids, and antispasmodic agents, either alone or in combination (8, 9). However, the side effects of analgesic drugs remain a major concern (10). The adverse effects of NSAIDs, which range from minor allergic reactions to kidney failure or gastrointestinal bleeding, may outweigh their benefits (11). In addition, the use of opioids is associated with the problems of abuse and elevation of ureteral pressure (12). Although analgesics such as acetaminophen is a safer option with less side-effects compared with NSAIDs and opioids, it could still cause complications such as weakness, elevated hepatic transaminases, or a low blood pressure (13). Therefore, a safe, effective, cost-effective, and simple approach free of pharmacological side effects is anticipated.

Recent meta-analyses have demonstrated the effectiveness of acupuncture for acute pain relief in patients with musculoskeletal disorders (14, 15). Through distinct neurohumoral and neurophysiological actions (16, 17), acupuncture plus moxibustion could be a more effective and safer therapeutic approach than routine treatments for non-specific low back pain (18, 19). Consistently, acupuncture is gaining popularity as an adjunctive therapy in the treatment of renal colic and for patients after extracorporeal shock wave lithotripsy (ESWL) (20–23). Although the clinical efficacy of acupuncture vs. analgesics for treating renal colic has been investigated in a recent systematic review (24), that report included studies focusing on conventional acupuncture as a monotherapy in a relatively small patient population so that the scope of acupuncture may be limited and the robustness of evidence may be impaired. Therefore, through incorporating the evidence from the latest randomized controlled trials (RCTs), the current meta-analysis attempted to elucidate the therapeutic efficacy of acupuncture compared to that of analgesics against renal colic with different indices for outcome assessment at different time points.

## 2. Materials and methods

This meta-analysis design followed the Preferred Reporting Items for Systematic Reviews and Meta-Analyses (PRISMA) 2020 statement and was registered with the International Center for Systematic Review of Prospective Registries (CRD42022346714).

### 2.1. Eligibility criteria

Studies that assessed the analgesic efficacy of acupuncture against renal colic were considered eligible. RCTs were selected based on the following criteria and predefined PICO (i.e., population, interventions, comparison, and outcome) framework: (a) Patient population: adult patients (≥18 years) with renal colic as the major clinical symptom; (b) Intervention: the use of acupuncture regardless of its location or type; (c) Comparison: the use of analgesics (e.g., NSAIDS, opioids) and antispasmodics through conventional routes, namely oral, intramuscular, or intravenous administration; (d) Outcomes: available information about response rate and results of pain severity assessment with visual analog scale (VAS) or any other numerical scales.

Exclusion criteria were: (1) non-RCTs; (2) studies which did not use acupuncture as the main therapeutic method; (3) those in which primary outcome information was missing; and (4) those combining acupuncture with analgesics, Chinese herbal medicine, or tuina.

### 2.2. Information sources and search strategy

The following databases were searched from their inception dates till July 15, 2022: MEDLINE, Cochrane CENTRAL register of controlled trials, Embase, Google scholar and the China National Knowledge Infrastructure (CNKI) database. The free texts and medical subject headings (i.e., MeSH terms in Medline) that were combined for searching included: (“renal colic” or “kidney stone” or “renal stone” or “urinary calculi” or “urinary calculus” or “ESWL” or “urolithiasis” or “nephrolithiasis” or “Ureteral calculi”) and (“Acupuncture” or “Electro acupuncture” or “Laser acupuncture” or “Needle acupuncture” or “auricular acupuncture”). For completeness of our literature search, review articles and the reference lists of the acquired studies were scrutinized for potentially eligible trials to be included in the current meta-analysis. We placed no restrictions on language, publication date, sample size, and country.

### 2.3. Selection process and data collection

Two independent reviewers were involved in the selection process and data collection. Prior to independent full-text review of the included research articles, each article’s title and abstract were screened to determine whether the study design, recipients, interventions, and outcomes were consistent with our study objectives for inclusion. Disagreements between the two reviewers were resolved through consulting with a third author. The following information was collected: first author, year of publication, gender distribution, patient’s age, sample size, drugs for conventional intervention, and country.

### 2.4. Outcomes and definitions of data items

The primary outcome was response rate which was defined as the percentage of participants with effective pain relief after intervention. The effectiveness of pain relief was in accordance with that defined in each study. The secondary outcome included the time of pain relief onset, pain score at 30–60 min, and the risks of side effects including dizziness, nausea, vomiting, and fatigue. The definition of time to pain relief was based on that in individual studies.

### 2.5. Risks of bias assessment

Two independent authors appraised the risks of bias of each included trial according to the Cochrane risk-of-bias tool for RCTs (RoB 2.0), which comprises five domains for assessment, namely selection of the reported results, deviations from intended interventions, outcome measurement, randomization process, and missing outcome (25). Only when a study showed a low risk of bias in all five domains could it be considered a “low risk” study, while a trial that exhibited “some concerns” in at least one domain was deemed to be having “some concerns.” A study having a high risk of bias in one or more domains was labeled as having an overall “high risk.”

### 2.6. Effect measures and data synthesis

The statistical approach to the present meta-analysis was as previously described (26–28). In detail, an outcome was obtained through merging of data from two or more studies. Efficacy of an intervention is expressed as effect size, which is shown as risk ratio (RR) with 95% confidence interval (CI) for a dichotomous variable and as mean differences (MD) for a continuous outcome. The Mantel–Haenszel random-effects model was adopted for the outcome analysis taking into consideration the heterogeneity in clinical and population-related parameters. The degree of heterogeneity, which was assessed with *I*^2^ statistics, was considered significant when it was > 50% (29). A leave-one-out sensitivity analysis was performed for an outcome showing *I^2^ >* 50% to determine the effect of the findings of each study. A funnel plot focusing on a specific outcome described in 10 or more datasets was inspected for symmetry to detect potential publication bias. Two-tailed tests were applied to all comparisons with a *p*-value below 0.05 being deemed statistically significant. Data synthesis was conducted with the Cochrane Review Manager (RevMan 5.3; Copenhagen: The Nordic Cochrane Centre), The Cochrane Collaboration. The significance of the association between age of the participants and response rate was examined with the Open Meta-Analyst software (Brown University, Providence, RI).

To avoid overlapping in the assessment of sample size in studies with multiple intervention arms, participants were divided according to the treatment arm that they belonged to as previously reported (30). While only the total number of participants could be divided without changing the means and SDs for continuous outcomes, both the event number and the total number of participants could be divided for dichotomous outcomes.

Taking into account the potential impact of false-positive results from multiple testing and sparse data on the study outcome, the robustness of the cumulative evidence derived from the current study was evaluated with trial sequential analysis (TSA) (TSA viewer version 0.9.5.10 Beta)^1^ as previously described (31). The relationship between the TSA boundary and the cumulative Z curve was scrutinized after the computation of the trial sequential monitoring and the required information size (RIS) boundaries. While a crossing of the cumulative Z curve over the TSA boundary denotes a sufficient level of evidence supporting the anticipated intervention effect without recourse to further studies, a failed interaction of the two represents inadequate evidence for a sound conclusion. For dichotomous outcomes, the RIS was computed with the setting of 5% type I error, 80% power, and a 20% relative risk reduction.

## 3. Results

### 3.1. Study selection and characteristics

**FIGURE 1:**
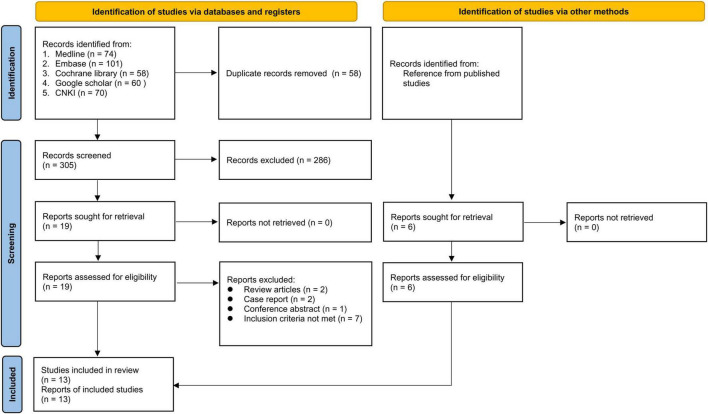
Flow diagram of study selection for the current meta-analysis.

Of the 363 potentially eligible articles identified through literature search, 58 were removed due to duplications. Of the remaining 305 records screened based on title and abstract, 286 were deemed ineligible. Further exclusion of 12 studies following full text screening of the 19 reports gave seven eligible RCTs (Figure 1). In addition, data extraction from a previous meta-analysis (24) provided six more eligible RCTs. Finally, 13 RCTs published from 1992 to 2021 with a total of 1,212 participants were included (12, 23, 32–42). All studies investigated the clinical efficacy of acupuncture vs. analgesics in the treatment of renal colic.

The characteristics of the included studies are shown in Table 1. The mean/median age of the participants ranged from 19 to 49 years in 11 studies (12, 23, 32–42), while two RCTs (34, 35) did not provide this information. The male prevalence ranged from 52 to 100% in ten studies (12, 23, 32, 33, 36, 38–42), while relevant detail was not provided in three studies (34, 35, 37) (Table 1). Details on acupoints and their anatomical locations as well as meridians and the definition of a positive response described in the included studies are summarized in Supplementary Table 1. In the control group, avafortan, acetaminophen, lornoxicam, tramadol, anisodamine, fortanodyn, scopolamine, bucinnazine, phenergan, atropine, acetaminophen, diclofenac, morphine, lornoxicam, or dolantin were used for comparison. Three studies (23, 41, 42) randomized patients into three groups, namely one intervention group (i.e., acupuncture) and two control groups (i.e., conventional treatment groups); therefore, comparison between the intervention and control groups required the splitting of the intervention group in the current study setting. The thirteen studies were conducted in four countries, namely, China (*n* = 10) (32–37, 39–42), Taiwan (*n* = 1) (38), Tunisia (*n* = 1) (12), and Turkey (*n* = 1) (23).

**TABLE 1:** Characteristics of studies (*n* = 13).

References	Age (years)^a^	N^a^	Male (%)	Intervention/control groups	Country
Lee et al. (38)	40.5 vs. 46.0	22 vs. 16	100	Acupuncture/Avafortan	Taiwan
Huang et al. (36)	19–58	25 vs. 27	63	Acupuncture/Phenergan, atropine	China
Kaynar et al. (23)	42.39 vs. 46.3	41 vs. 80	63	Acupuncture/Acetaminophen, diclofenac	Turkey
Beltaief et al. (12)	42 vs. 41.8	54 vs. 61	53	Acupuncture/Morphine	Tunisia
Zhang et al. (39)	47.95 vs. 49.0	39 vs. 41	52	Acupuncture/Lornoxicam	China
Huang and Li (40)	25 vs. 23	30 vs. 30	58	Body and auricular acupuncture/Dolantin	China
Lin et al. (41)	36.6 vs. 36.0	90 vs. 90	53	Eye acupuncture/Morphine, bucinnazine	China
Ju and Niu (42)	39 vs. 39	80 vs. 160	70	Acupuncture/Dolantin, scopolamine	China
Qiu et al. (33)	26.7 vs. 28.3	30 vs. 30	66	Acupuncture/Tramadol, atropine	China
Xiang et al. (34)	NA	60 vs. 60	NA	Acupuncture/Anisodamine, tramadol	China
Xiao (37)	39 vs. 39.6	20 vs. 20	NA	Acupuncture/Flurbiprofen axetil	China
Xu et al. (32)	28.32 vs. 27.26	25 vs. 27	62	Acupuncture/Fortanodyn	China
Yang et al. (35)	NA	27 vs. 27	NA	Acupuncture/Fortanodyn	China

^a^Present as intervention vs. control groups; NA, not available.

### 3.2. Risk of bias assessment

**FIGURE 2:**
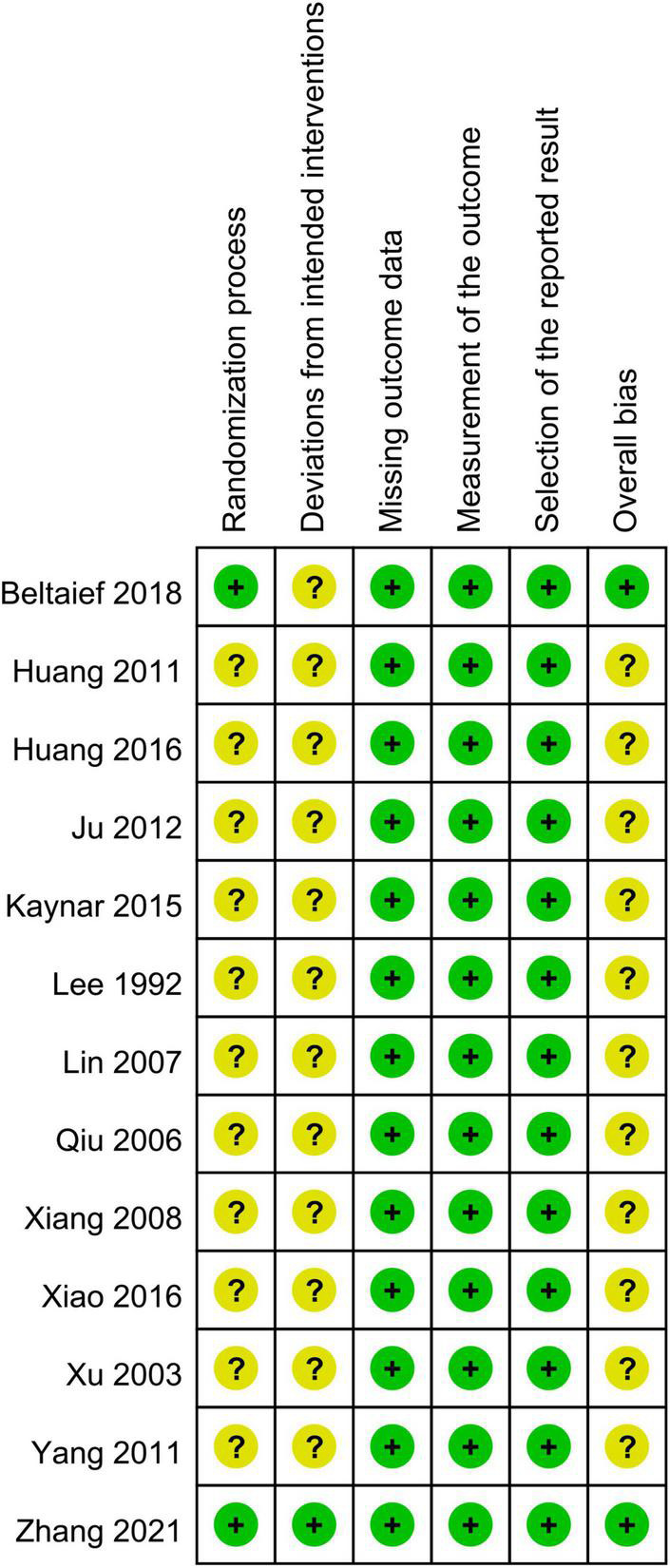
Summary of risks of bias. Green, low risk; yellow, some concerns.

Results of the risk of bias assessment are shown in Figure 2. The risks of bias for the randomization process and deviations from intended interventions were judged to be having “some concerns” in 11 studies and 12 studies, respectively (Figure 2). The overall bias was rated as “low” and “some concerns” in two and 11 studies, respectively.

### 3.3. Results of syntheses

#### 3.3.1. Primary outcome: Response rate

**FIGURE 3:**
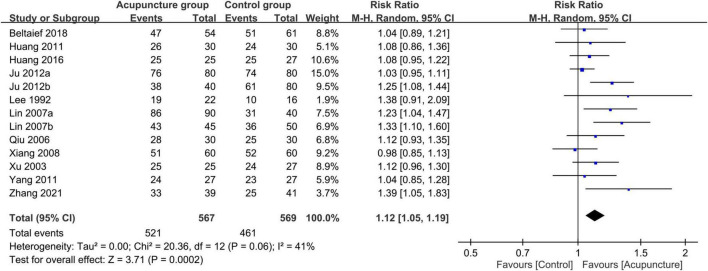
Forest plot comparing response rate between acupuncture and control groups. RR, risk ratio; M-H, Mantel–Haenszel; CI, confidence interval.

**FIGURE 4:**
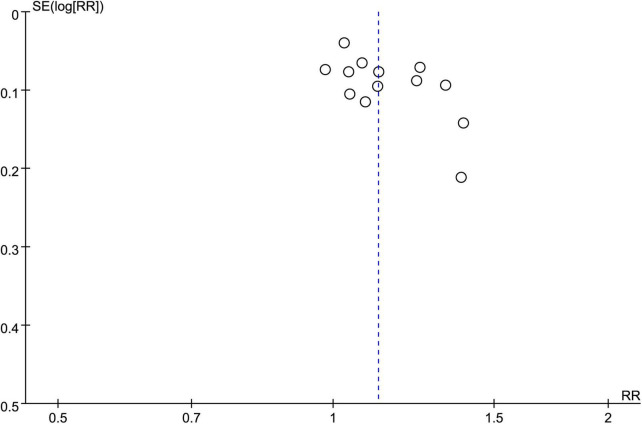
Funnel plot on response rate indicating a low risk of publication bias.

**FIGURE 5:**
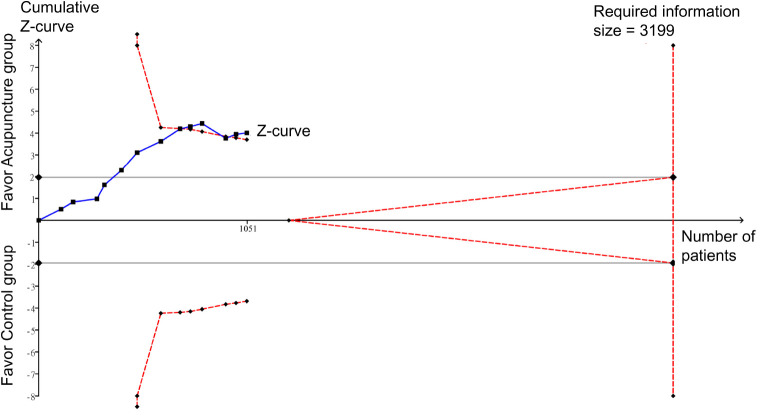
Trial sequential analysis of the robustness of evidence in support of the significant difference in response rate between intervention and control groups.

**FIGURE 6:**
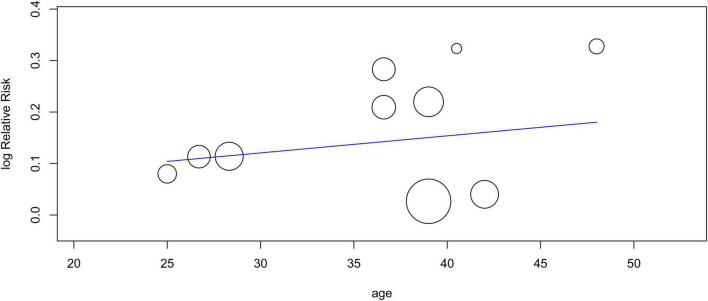
Meta-regression plot showing the association between patient age and response rate.

Eleven studies involving 1,136 patients (acupuncture group: 567 patients, control group: 569 patients) were available for response rate analysis. The results of the meta-analysis showed a significantly higher efficiency of acupuncture for alleviating renal colic than that in the controls (RR = 1.12, 95% CI: 1.05–1.19, *p* = 0.0002, *I*^2^ = 41%) (Figure 3). Funnel plot indicated a low risk of publication bias (Figure 4). A sufficient level of evidence supporting this finding was reflected by crossing of the cumulative Z-curve over RIS on TSA, indicating a robust conclusion for our primary outcome (Figure 5). Meta-regression analysis demonstrated no correlation between age and response rate **(***p* = 0.549**)** (Figure 6).

#### 3.3.2. Secondary outcomes

**FIGURE 7:**
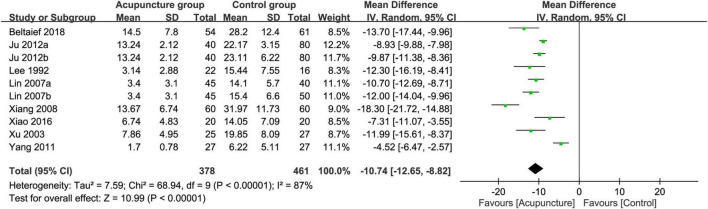
Forest plot comparing the time to pain relief between acupuncture and control groups. CI, confidence interval; IV, inverse variance.

**FIGURE 8:**
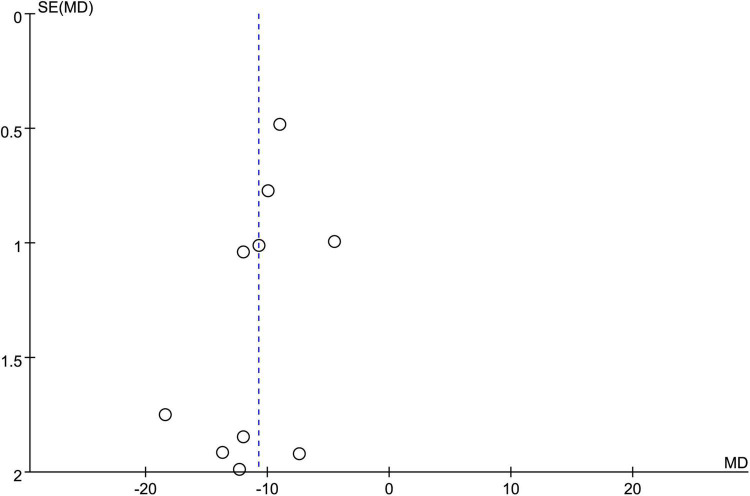
Funnel plot on the time to pain relief showing a low risk of publication bias.

**FIGURE 9:**
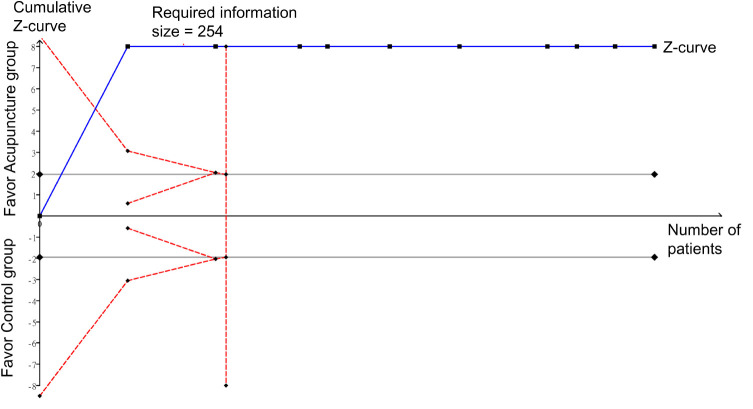
Trial sequential analysis of the robustness of evidence supporting the difference in time to pain relief between the acupuncture and control groups.

Merged results of the eight studies with available information for analysis showed a faster pain relief in the acupuncture group compared with the control group (MD = −10.74 min, 95% CI: −12.65 to −8.82, *p* < 0.00001, *I*^2^ = 87%, 839 patients) (Figure 7). Funnel plot demonstrated a low risk of publication bias (Figure 8). Crossing of the cumulative Z-curve over the RIS on TSA suggested sufficient evidence to reach a firm conclusion (Figure 9).

**FIGURE 10:**
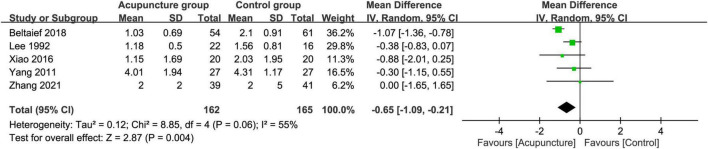
Forest plot comparing pain score at 30–60 min between acupuncture and control groups. CI, confidence interval; IV, inverse variance.

**FIGURE 11:**
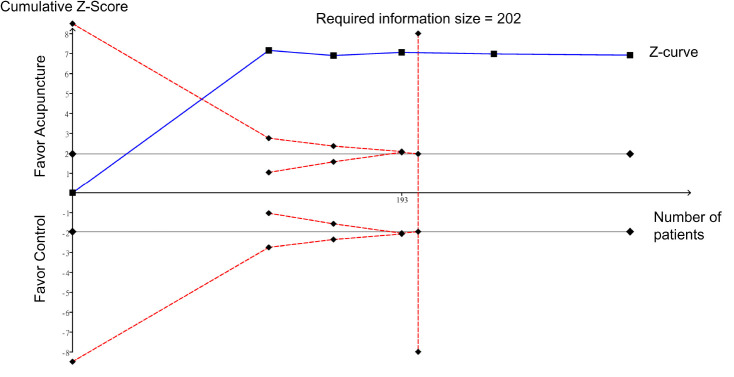
Trial sequential analysis of the weight of evidence supporting the difference in pain score at 30–60 min between the acupuncture and control groups.

Analysis of five RCTs that provided relevant details for the analysis of pain score at 30–60 min also revealed a significant low pain score in the acupuncture group compared to that in the control groups (MD = −0.65, 95% CI: −1.09 to −0.21, *p* = 0.004, *I*^2^ = 55%) (Figure 10). Sufficient evidence of this finding was endorsed by crossing of the cumulative Z-curve over the RIS on TSA (Figure 11).

**FIGURE 12:**
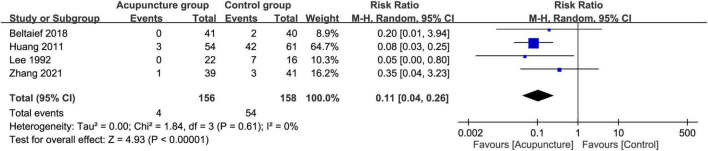
Forest plot comparing the overall risk of side effects between acupuncture and control groups. CI, confidence interval; RR, risk ratio; M-H, Mantel–Haenszel.

Meta-analysis showed that the use of acupuncture was associated with a lower overall risk of side effects compared to that pertinent to the use of analgesics (RR = 0.11, 95% CI: 0.04–0.26, *p* < 0.0001; four RCTs; *n* = 314) (Figure 12). The acupuncture-related side effects of the four studies that provided relevant information are summarized in Supplementary Table 2.

## 4. Discussion

Renal colic, which is often presented as acute excruciating pain that needs urgent medical attention, is the most common reason for emergency department visits for those with ureterolithiasis. Our findings not only demonstrated the effectiveness of acupuncture for pain relief in this clinical condition but also revealed a rapid onset of its analgesic action and a lower pain score at 30–60 min compared to those receiving conventional analgesic treatments. Moreover, our results showed a lower risk of complications associated with the acupuncture approach. In addition, TSA endorsed the robustness of evidence in support of the analgesic effectiveness of acupuncture with a relatively short time of action onset and low pain score at 30–60 min. Furthermore, our findings demonstrated no association between the age of participants and the analgesic efficacy of acupuncture in this clinical setting.

NSAIDs and opioids, when used alone or in combination, are the main options for the treatment of renal colic (43). Moreover, acute pain can usually be relieved by intravenous or intramuscular administration of centrally acting analgesics, such as morphine (44), diclofenac (45), dolantin and lornoxicam. Nevertheless, satisfactory pain relief could not be attained in about 16–42% of patients who sought other analgesic rescue measures (46). Although the mechanism underlying acupuncture-induced analgesia is not clear, it has been shown to exert its analgesic effects though nervous system modulation (47–49), endocrine regulation (50–52), and smooth muscle relaxation (53, 54) on visceral pain. With respect to the analgesic effect of neuropathic pain, electroacupuncture might alleviate the hyperalgesia state through inhibition of prostaglandin E2 secretion after 1 week of intervention in a rat model (55). Labor pain of rats has been reported to be relieved after acupuncture point treatments via κ-opioid receptor activation and enhanced prodynorphin protein expression in the lumbar spinal cord (55). Liu et al. found that acupuncture on ST25 and ST37 points could reduce the colonic concentrations of 5-hydroxytryptamine (5-HT) in chronic visceral hypersensitivity rats (56). A retrospective study showed that hospitalized patients with urinary retention receiving acupuncture significantly achieved a reduction in residual urine volume (PVR) with 87.6% being cured of their urinary retention (57).

The main finding of the current systematic review of 13 RCTs was the higher response rate of acupuncture compared to conventional interventions in patients with renal colic. Consistently, several previous systematic reviews have reported superior outcomes associated with acupuncture in the treatment of chronic low back pain (14), acute pancreatitis (58), primary dysmenorrhea (59), and knee osteoarthritis (60) compared to those in the pain medication or sham acupuncture groups. In the setting of renal colic, prompt and effective pain relief is critical for avoiding alternative and rescue treatments as well as reducing the total length of stay in the emergency department (61). A combination of acupuncture with conventional treatment has been shown to substantially reduce the length of hospital stay and the recovery time from the first bowel movement in patients diagnosed with acute pancreatitis (58).

Our result on the response rate was also consistent with that of a recent meta-analysis (24). In contrast with that systematic review that included eight studies that used acupuncture as a monotherapy in the intervention group and the other eight that included acupuncture as an adjuvant therapy in the intervention group (24), one of the merits of the current meta-analysis was the inclusion of more studies (i.e., 13 RCTs) focusing on acupuncture as a monotherapy with the exclusion of studies that used acupuncture as an adjuvant therapy to minimize the potential bias. In fact, the previous meta-analysis demonstrated no improvement in response rate when a combined regimen of acupuncture and conventional medications was compared with conventional medical treatments among patients with renal colic (24).

The current meta-analysis demonstrated that the onset of pain relief with acupuncture was faster than that associated with conventional analgesics (i.e., MD = −10.74 min). Our finding was consistent with that of a previous paired meta-analysis that supported the prompt analgesic effect of acupuncture for acute pain relief in a variety of settings with an onset ranging from 15 to 30 min (61). Similarly, a 2021 meta-analysis of the immediate analgesic effect of acupuncture against acute low back pain reported a better immediate analgesic effect of acupuncture than conventional analgesics (14). A previous randomized double-blind clinical trial (62) also showed that acupuncture provided faster pain relief compared to ibuprofen in patients with acute toothache. Regarding the possible mechanisms, previous experiments using a male rat model of acute adjuvant arthritis male demonstrated that acupuncture could trigger immediate analgesia through boosting β-endorphin concentration in the cerebrospinal fluid (63, 64).

Besides a prompt onset, our findings showed a persistent analgesic effect of acupuncture comparable to that of analgesics as long as 30–60 min after intervention. Morphine is the most potent agent among the other analgesics used for acute renal colic. Despite the fast onset of its analgesic action (i.e., 5–15 min intravenously, 1 h orally), its duration of action is only 3–6 h in patients with acute limb traumatic injury (65). In contrast, in addition to an early onset of acupuncture-induced analgesia at 15–30 min as reported in previous systematic reviews on patients with musculoskeletal pain (48, 61), the analgesic effect can persist for up to 3 days (16). The onset of acupuncture-triggered analgesia, which is manifested as a feeling of soreness, numbness, heaviness, and distension at the site of acupuncture, signifies activation of pain-related regions of the brain (66). Taking into account the comparatively fast onset as well as a long duration of action, acupuncture may be considered an alternative analgesic strategy in the treatment of acute renal colic.

Compared to the well-known side effects of NSAIDs such as gastrointestinal complications (e.g., bleeding, ulcer perforation) (60), the relative safety of acupuncture and its association with few serious adverse events render it a promising option for patients with pain not responding to regular medications and those experiencing serious drug-related side effects. Our systematic review demonstrated a lower risk of complications in patients receiving acupuncture compared to those subjected to conventional interventions. Adverse events associated with acupuncture including needle-site pain, nausea and vomiting, syncope or dizziness (67) are rare (11) with an overall incidence below 0.1% (67). Such minor discomforts seem to disappear spontaneously within minutes or hours (68).

Two concerns should be addressed in current meta-analysis. First, the efficacy of acupuncture may vary with the experience of the acupuncturist including the choice of acupoints and procedural skills. Nevertheless, despite the demonstration of considerable variation in the localization of acupoints among acupuncturists in a prior report (69), another study did not show a significant impact of the degree of expertise of acupuncturists on treatment efficacy (70). Therefore, provided that such information was not available in our included studies, their universal demonstration of “Deqi” as a positive response (Supplementary Table 1) suggested an effective acupuncture that theoretically validated a comparison of their treatment outcomes. Second, despite previous reports of tolerance of habituation of acupuncture in an animal experimental setting (71, 72) attributable to an interaction between an accelerated release of central cholecystokinin octapeptide and endogenous opioid peptides (73, 74), such a phenomenon was not described in our included studies. Further clinical investigations are warranted to address this issue.

The strengths of this study could be summarized as follows: (1) In addition to the study of the efficacy of conventional manual acupuncture for treating renal colic as in a recent meta-analysis (24), the present investigation included other acupuncture techniques (e.g., electro-acupuncture) in our analysis that involved more than a thousand participants; (2) Compared with the previous meta-analytic study (24), we also examined the robustness of evidence and heterogeneity by using TSA and meta-regression analysis, respectively; and (3) In contrast to the results of the prior meta-analysis that demonstrated a large publication bias (24), a low level of publication bias in this study may reflect a better quality of evidence.

On the other hand, this study had several limitations. First, most studies were from China (i.e., 10 out of 13) so that the results of this study may not be extrapolated to populations of different ethnic backgrounds. Second, the sample sizes in a number of our included studies were relatively small (i.e., eight studies each with less than a 100 participants). Third, most studies provided little or no description regarding their randomization, allocation concealment, or blinding processes. Fourth, because most studies only used a single parameter (i.e., response rate) as the final outcome indicator, the subjective nature of the parameter may affect the reliability of the final results. Objective outcome indicators, such as time to complete pain relief, episodes of renal colic during treatment and adverse effects as well as circulating prostaglandin E2 concentration, may be included in future studies to provide more accurate information for elucidating the beneficial effects of acupuncture in this clinical setting. Fifth, heterogeneity from the use of different anti-inflammatory and antispasmodic agents for pain relief may contribute bias to our outcomes. Finally, the lack of information about long-term follow-ups after treatment precluded our analysis of the long-term efficacy as well as the risk of recurrence after interventions.

## 5. Conclusion

The results of the current meta-analysis showed that the clinical efficacy of acupuncture for renal colic was superior to that of conventional analgesics with a rapid onset of analgesia and relatively few adverse effects. However, due to the overall small number of patients included in the present study, more multicenter, large-scale clinical trials with more objective study designs are needed to validate the results of this study.

## Data availability statement

The original contributions presented in this study are included in the article/Supplementary material, further inquiries can be directed to the corresponding author.

## Author contributions

H-TC and C-FK: conceptualization and literature search. C-CH: methodology. L-CL and A-CC: trial selection. K-CH: data analysis. H-TC and C-CH: data extraction. K-CH and H-TC: writing—original draft preparation. H-TC and C-KS: writing—review and editing. All authors have read and agreed to the published version of the manuscript.

## Footnotes

1
www.ctu.dk/tsa
