# Supplementary material for: Clinical efficacy of acupuncture for pain relief from renal colic: A meta-analysis and trial sequence analysis
Source: Front Med (Lausanne). 2023 Jan 9;9:1100014. doi: 10.3389/fmed.2022.1100014 (PMC9868182; doi:10.3389/fmed.2022.1100014)
Supplement: Supplementary file 1 [file Table_1.docx]

**Supplemental Table 1.** Detail of needling in the included studies (n = 13)

| Author (year) | Acupoints and anatomical locations | Meridians | Response sought |
| --- | --- | --- | --- |
| Lee (1992) [38] | Extra points loin and leg (EX-UE 7) on the hand, BL-21 through BL-25, BL-45 through BL-47, or Yao-Yen (EX-B7) on the back | Bladder Meridian (BL) | Deqi |
| Huang (2016) [36] | BL-23 on the back, BL-45 and ST-36 on the leg | Bladder Meridian (BL)  and Stomach Meridian (ST) | Deqi |
| Kaynar (2015) [23] | BL-21 through BL-24, BL-45 through Ll-48 on the back and leg | Bladder Meridian (BL) | Deqi |
| Beltaief (2018) [12] | BL-21 through BL-26, BL-45 through BL-49 on the back and leg | Bladder Meridian (BL) | Deqi |
| Zhang (2021) [39] | SP-6 and SP-9 | Spleen Meridian (SP) | Deqi |
| Huang (2011) [40] | SP-6 and GB-34 on the leg, Shenmen (TF 4), Kidney (CO 10), and Bladder (CO 9) on ears | Gallbladder Meridian (GB), Stomach Meridian (ST) and Ear points | Deqi |
| Lin (2007) [41] | Eye acupuncture | Eye acupuncture | Deqi |
| Ju (2012) [42] | PC-6 on the hand and ST-36 on the leg | Pericardium Meridian (PC) and Stomach Meridian (ST) | Deqi |
| Qiu (2006) [33] | ST-36 on the leg | Stomach Meridian (ST) | Deqi |
| Xiang (2008) [34] | BL-23 on the back, BL-45 and ST-36 on the leg | Bladder Meridian (BL)  and Stomach Meridian (ST) | Deqi |
| Xiao (2016) [37] | BL-23 on the back | Bladder Meridian (BL) | Deqi |
| Xu (2003) [32] | BL-23 on the back, BL-45 and ST-36 on the leg | Bladder Meridian (BL)  and Stomach Meridian (ST) | Deqi |
| Yang (2011) [35] | KI-2 and KI-3, BL-60 and BL-63 on the leg | Kidney Meridian (KI) and Bladder Meridian (BL) | Deqi |
