# Supplementary material for: Clinical efficacy of acupuncture for pain relief from renal colic: A meta-analysis and trial sequence analysis
Source: Front Med (Lausanne). 2023 Jan 9;9:1100014. doi: 10.3389/fmed.2022.1100014 (PMC9868182; doi:10.3389/fmed.2022.1100014)
Supplement: Supplementary file 2 [file Table_2.docx]

**Supplemental Table 2.** Expected side effects of acupuncture in the included studies

| Author (year) | Expected side effects | Actual event/ Number of patients |
| --- | --- | --- |
| Beltaief (2018) [12] | Local rash/bleeding, itching, needle blockage, and fainting | 0/41 |
| Huang (2011) [40] | Local pain | 3/54 |
| Lee (1992) [38] | Mentioned but not specified | 0/22 |
| Zhang (2021) [39] | Frequent urination after acupuncture relieved two hours later | 1/39 |
